# Behavioral and Functional Brain Activity Alterations Induced by TMS Coils with Different Spatial Distributions

**DOI:** 10.1523/ENEURO.0287-22.2023

**Published:** 2023-04-12

**Authors:** Gaby S. Pell, Yiftach Roth, Hamutal Shachar, Moshe Isserles, Noam Barnea-Ygael, Abraham Zangen

**Affiliations:** 1 Department of Life Sciences and the Zlotowski Centre for Neuroscience, Ben-Gurion University of the Negev, Beer-Sheva, 84105, Israel; 2 BrainsWay Ltd., Jerusalem 9777518, Israel; 3 Department of Psychiatry, Hadassah-Hebrew University Medical Center, Jerusalem, 91120, Israel

**Keywords:** electric fields, fMRI, neurostimulation, prefrontal cortex, reward, transcranial magnetic stimulation

## Abstract

Previous investigation of cognitive processes using transcranial magnetic stimulation (TMS) have explored the response to different stimulation parameters such as frequency and coil location. In this study, we attempt to add another parameter by exploiting the spatial profiles of TMS coils to infer regional information concerning reward-related behavior. We used different TMS coils to modulate activity in the prefrontal cortex (PFC) and examined resulting changes in behavior and associated brain activity. More specifically, we used the Figure-8 coil to stimulate a portion of the dorsolateral PFC (DLPFC) and the H-Coil to stimulate a larger volume within the lateral PFC (LPFC). Healthy human volunteers completed behavioral questionnaires (*n* = 29) or performed a reward-related decision-making functional MRI (fMRI) task (*n* = 21) immediately before and after acute high-frequency stimulation (10 Hz) with either a Figure-8 coil, H-Coil, or a sham coil. Stimulation was found to induce behavioral changes as well as changes in brain activation in key nodes of the reward network. Right LPFC, but not right DLPFC or sham, stimulation was found to induce changes in both behavioral scores and brain activation in key nodes of the reward system. In conclusion, this study supports the role of the right LPFC in reward-related behavior and suggest that the pathways through which the observed effects were generated are located outside the area of the DLPFC that is traditionally targeted with TMS. These results demonstrate the use of TMS coils with different spatial profiles as an informative tool to investigate anatomic and functional correlates of behavior.

## Significance Statement

When trying to associate cognitive function with brain anatomy, probing with neuromodulation has emerged as a useful approach. One can modulate brain activity with techniques such as transcranial magnetic stimulation (TMS) and examine the effect on behavior. Yet, hypotheses often associate behavior with relatively large brain areas which is inefficient, requiring many experimental groups to provide useful information. Here, we describe an approach using TMS coils with different field distributions to achieve a similar goal with reduced time and simplified resources. Our results indicated a pattern that differed between a focal coil (Figure-8) coil and a wider/deeper coil (H-Coil). Future studies may localize the origin within the frontal cortex that drives these effects, and thereby further establish the association between structure and function.

## Introduction

A consistent finding across imaging studies of value-based learning and decision-making is the prominent involvement of the lateral prefrontal cortex (LPFC; Dixon and Christoff, 2014). Indeed, the LPFC, and especially the dorsolateral PFC (DLPFC) have been implicated in the pathogenesis of several psychiatric and neurologic disorders with affected reward-related behavior, including schizophrenia, anxiety, and posttraumatic stress disorder (Hopper et al., 2008; Maresh et al., 2014; Subramaniam et al., 2015; Park et al., 2016; Pu et al., 2016; Zhang et al., 2016). Moreover, there is evidence for the efficacy of transcranial magnetic stimulation (TMS) over the PFC for the treatment of other conditions with impaired reward-related behavior such as depression, addictions, Alzheimer’s disease, schizophrenia, and eating disorders (Fox et al., 2014; Moeller et al., 2022). These effects are attributed to the technique’s ability to redress imbalances in the excitability of brain networks and neurotransmitter concentrations that characterize these conditions (Pell et al., 2011). Notably, the United States Food and Drug Administration (FDA) has approved the use of TMS over the DLPFC for the treatment of major depression, a disorder in which anhedonia, believed to result from impaired processing in the brain’s reward system, is a hallmark feature (APA, 2013). In fact, FDA approval has been given to two different classes of TMS coils, the Figure-8 and the H-Coil, following two multicenter trials (O’Reardon et al., 2007; Levkovitz et al., 2015). While the former generates a relatively superficial and focal effective electric field, the later induces a deeper and more widespread field (Roth et al., 2007; Rosenberg et al., 2010; Deng et al., 2013; Alyagon et al., 2020; Zibman et al., 2021). The differences in the depth-focality trade-off between the coils translate to a different volume of tissue being stimulated under each coil.

While the aforementioned effects of TMS were achieved in pathologic populations following repeated sessions, several studies have shown that acute TMS, when applied over the PFC of healthy individuals, affects specific reward-related behaviors (Levasseur-Moreau and Fecteau, 2012; Cho et al., 2015). A frequency dependence for this effect has been reported so while acute high-frequency stimulation over the left DLPFC increases responsiveness to rewarding stimuli in healthy subjects (Ahn et al., 2013), low-frequency stimulation over the right DLPFC lead to riskier decision-making (Knoch et al., 2006; Ahn et al., 2013). These observations complement previous investigations of cognitive processes using TMS, which have employed a variety of different stimulation parameters such as frequency, coil location, and dosage (Beynel et al., 2019).

A recent publication showed that rTMS with a focal Figure-8 coil differentially affects distinct clusters of symptoms in MDD patients depending on the placement of the coil (Siddiqi et al., 2020). While this finding is an important step in understanding the mechanism behind clinical rTMS, it also presents a tremendous challenge. Stimulating the target for each symptom cluster would require additional sessions to the current treatment protocol to treat each cluster in series. Alternatively, a larger coil that stimulates all cluster targets simultaneously was suggested by the authors to be more effective at poly-symptomatic treatment by uniformly modulating multiple clusters. They used clinical depression data from depression studies using the H-Coil and the Figure-8 to provide evidence for this claim.

Here, we set out to investigate whether the modified spatial distributions of the electric fields between the different coils can be further exploited to shed light on the localization of cognitive processes. More specifically, we used the Figure-8 coil to stimulate a portion of the DLPFC and the H-Coil to stimulate a larger volume within the LPFC. We hypothesized that coil-related differences will be evident both at a behavioral level and in the pattern of brain activity during reward-related tasks. To test this, a design was employed that separately examined functional and behavioral changes between measurements taken before (PRE) and immediately following (POST) acute stimulation. The novel study design offers the advantage of being able to relate findings back to the underlying anatomy and to shed light on regional specificity within the prefrontal cortex.

## Materials and Methods

### Procedure

**Figure 1.**
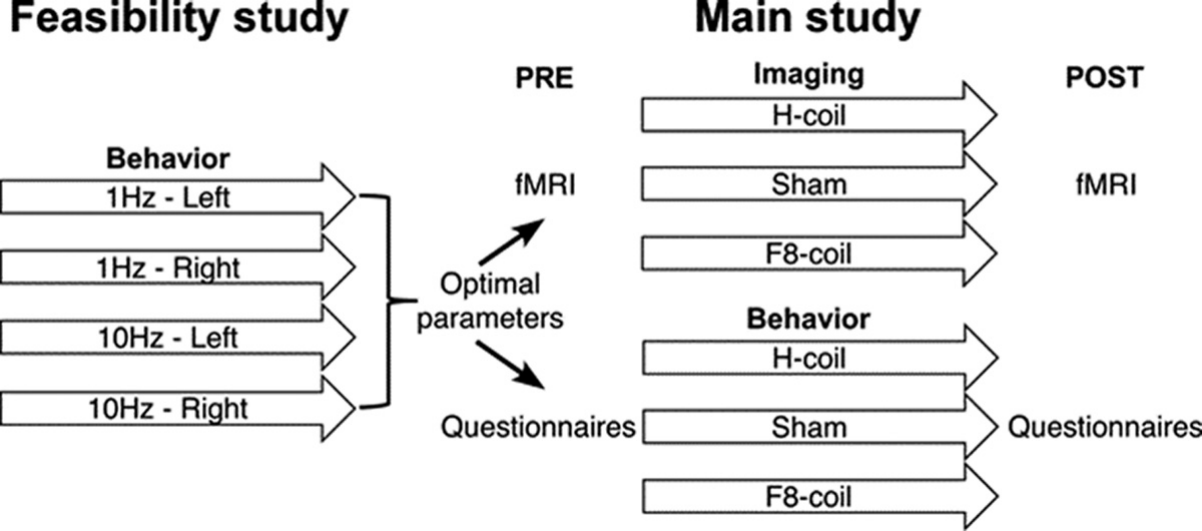
The study followed a two-stage trial design. The parameters for optimal induction of motivational alterations were determined in a feasibility study. These parameters were implemented in the imaging and behavioral parallel arms of the main study.

The study design is summarized in Figure 1. The study included a feasibility component that aimed to determine the optimal choice of stimulation frequency (1 or 10 Hz) and location (left or right LPFC) for the effective induction of alterations in motivation. The main experiment employed the obtained parameters to investigate the neuronal correlates and behavioral consequences of alterations induced by acute stimulation with the different coils. This experiment included an functional MRI (fMRI) arm, in which subjects performed the Iowa Gambling Task (IGT) task inside the scanner, and a behavioral arm, in which questionnaire were completed to evaluate motivation [motivational VAS (mVAS); Stubbs et al., 2000; Gorwood et al., 2015], affect [positive and negative affect schedule (PANAS); Watson et al., 1988], or both [modified achievement goal questionnaire (AGQ); Elliot and Sheldon, 1997]. It should be noted that different sets of subjects were used in each stage of the experiment and for the behavioral and neuroimaging studies.

### Subjects

TMS-naive volunteers (*n* = 89 participants; age: 24.2 ± 5.48 year; mean ± SD; 36 females) were recruited through advertising. All subjects were healthy with no history of psychiatric or neurologic diseases, and the different subgroups were stratified by age and gender using a computer program (Interactive Web Randomization System; Medpace’s ClinTrak). Before participation, all subjects signed an informed consent form and declared the absence of known TMS contraindications. Subjects were monetarily compensated ($30) for their time, and those who completed the fMRI decision-making task were given an additional payment according to their performance (up to $20). The study was approved by the local Institutional and National Review Board and was performed in accordance with the most recent version of the Declaration of Helsinki.

### Transcranial magnetic stimulation

**Figure 2.**
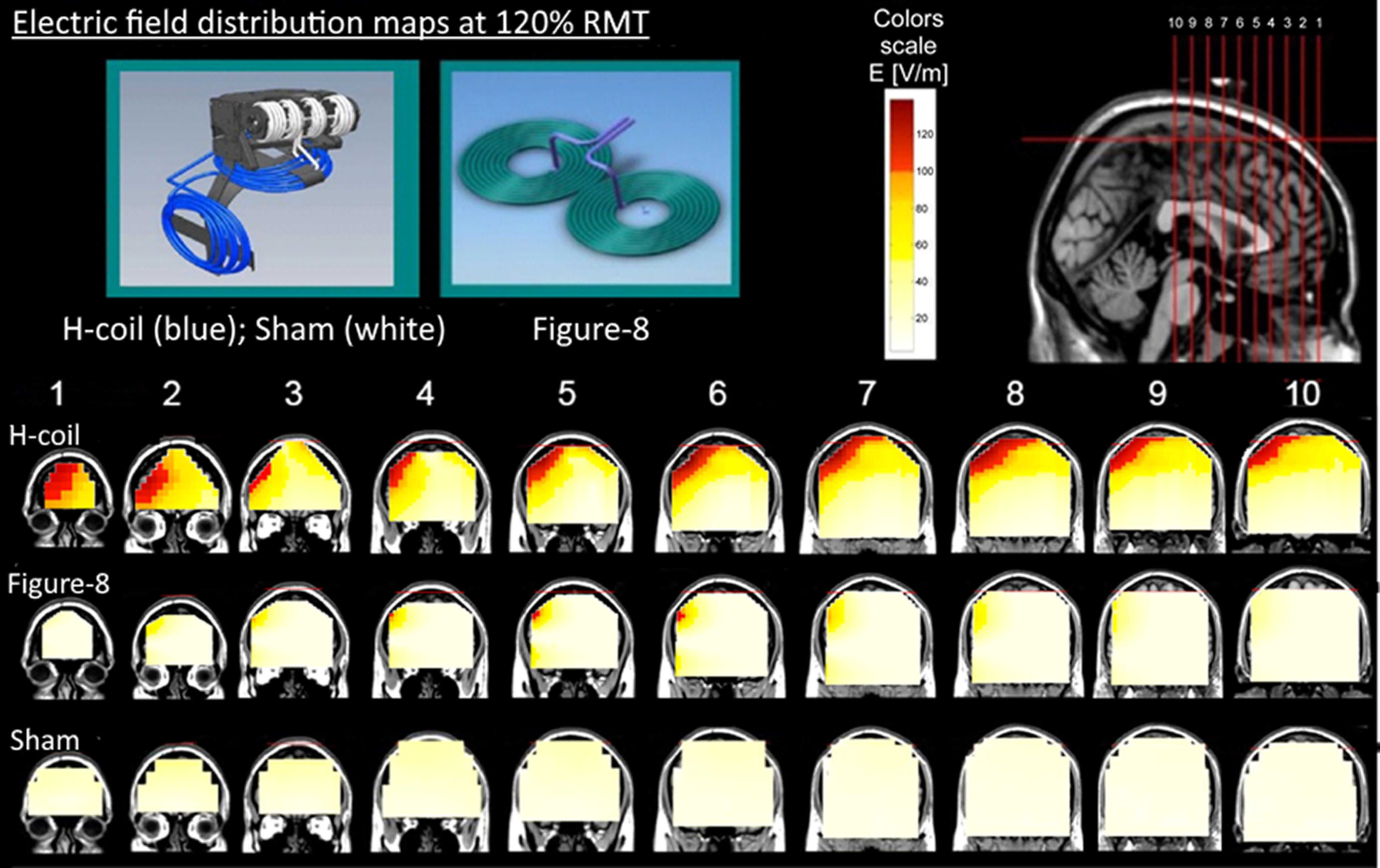
Electric field distributions induced by the TMS coils when placed over the right DLPFC, obtained in a phantom and overlaid on MRI images. The colored maps describe the absolute magnitude of the electric field for the various TMS coils. These were measured in a phantom model of the human head with an equivalent stimulation amplitude to that used in this study (i.e., stimulator output equivalent to 120% of an average motor threshold). In the color scale, red indicates a field magnitude above neural activation threshold (100 V/m), while white and yellow indicate field magnitude below the threshold. In the top left inset, the H-Coil is illustrated by the blue wire, while the sham coil is illustrated by the white wire wound on a cylindrical former. Anatomical images are shown in radiologic coordinates.

TMS was delivered with a Magstim Rapid^2^ stimulator (Magstim) using three types of coils. The H6-coil (BrainsWay), designed according to the principles of deep TMS (Roth et al., 2002; Tendler et al., 2016; Alyagon et al., 2020) to convey a deep and widespread stimulation to the target area. The coil was air-cooled and was integrated within a helmet that attached firmly to the head. A sham TMS coil (BrainsWay), based on a toroidal winding, integrated within the same helmet as the H-Coil, which induced similar acoustic sensations but only negligible cortical electric field. An air-cooled Figure-8 coil (Magstim) that was attached to a standard gantry. A sketch of the coils and distribution maps of the induced electric fields are shown in Figure 2.

In accordance with the results of the feasibility trial, stimulation sessions consisted of high-frequency repetitive TMS (rTMS; 10 Hz, 2-s trains, 20-s intertrain interval) applied to the right PFC. In each session, 900 pulses were administered at an intensity of 120% of RMT, determined as the minimum stimulation output that induced 50% chance of visual thumb abduction (Pridmore et al., 1998). The target area, for all coils, was defined as the region 6 cm anterior to the primary motor hand area (M1; Herbsman et al., 2009; Johnson et al., 2013). Orientation of the Figure-8 coil was along the standard posterior-lateral direction (i.e., 45° with respect to the sagittal direction).

Sources of potential variability include subjective subject comfort during the stimulation, which may affect behavioral and functional MRI results. In order to compare this factor, subjects were asked to rate their overall feelings following the stimulation.

### Neuroimaging

Stimulation-induced changes in neuronal activity were evaluated using a reward-related decision-making fMRI task [see below, Iowa Gambling Task (IGT)] that was executed PRE and POST stimulation with either the H-Coil, the Figure-8 coil, or the sham coil (*n* = 21 subjects, 7 in each group). To minimize the interval between the end of the stimulation session and the beginning of the second imaging session, the stimulation was performed in a room adjacent to the fMRI scanner and the subjects were returned to the scanner as quickly as possible after stimulation (<5 min).

#### Functional MRI

A Siemens 3T Trio MRI system (Siemens) was used together with a 32-channel RF coil. The parameters of the fMRI sequence were adjusted to minimize potential imaging artefacts in areas that were expected to be activated by the decision-making task (Deichmann et al., 2003). An initial pilot study was performed to determine the optimal parameters (data not shown). The following parameters were used for the gradient echoplanar imaging (EPI) BOLD sequence: TE = 25 ms, TR = 2000 ms, image matrix = 64 × 64, in-plane resolution = 3 × 3 mm, slice thickness = 3 mm with 1 mm gaps between slices, number of slices = 36, FOV = 192 mm, bandwidth = 220 kHz, volumes per scan = 405, duration of scan = 13.5 min. The slices were rotated from the transverse toward the coronal plane by 30° relative to the AC-PC line to reduce the influence of in-plane susceptibility gradients. The PRE scanning session comprised a localizer, a high-resolution anatomic scan (MP-RAGE sequence, TR/TE/TI = 8/4/1000 ms, image matrix = 256 × 256 × 70, resolution = 1 × 1×1 mm) and then the first functional run. The POST scanning session comprised a rapid localizer scan that was immediately followed by the second functional run.

Regions of interest (ROIs) were created in the targeted area (“target ROI”) and in task-related areas (“task-related ROIs”). The target ROI was defined as the supra-threshold electrical field induced by the Figure-8 coil (Fig. 2), while the task-related ROIs selected major nodes of the reward network, the orbitofrontal cortex (OFC), anterior cingulate cortex (ACC), and the insular cortex (Bush et al., 2002; McClure et al., 2004; Haber, 2011; Albrecht et al., 2013). The pregenual ACC (pgACC) within Brodmann area 32 was selected as it has been strongly implicated in reward processing and subjective emotional state (Cho and Strafella, 2009; Dixon and Christoff, 2014). Brodmann area 11 was used to create an ROI in the OFC and Brodmann area 13 was used to create an ROI in the insular cortex.

#### Iowa Gambling Task (IGT)

The subjects performed the task inside the scanner, PRE and POST stimulation. This probabilistic reward-related task was designed to be a simulation of real-life decision-making and was chosen for this study since it activates a wide range of brain areas involved with executive functions and reward-processing (Bechara et al., 2000; Li et al., 2010; Alexopoulos et al., 2015) that may overlap with the applied TMS field.

The goal of the task is to maximize profit. In brief, starting from an initial allocation of cards, subjects are required to make a series of card selections from one of four card decks (A, B, C, and D). Each selection is followed by the presentation of a reward and a penalty. Decks A and B are considered the disadvantageous decks because they yield high immediate rewards but higher long-term penalties and, consequently, a loss in the long-term. Decks C and D are the advantageous decks as they yield low immediate rewards but smaller long-term penalties, which, therefore, result in a long-term gain. The task was previously adapted for fMRI (Lin et al., 2008) and was programmed for the current study using the Presentation software package (Neurobehavioral Systems). The simple block design approach was selected to maximize the amplitude of the task-induced signal changes. The task comprised five blocks of the reward-based task interleaved with the same number of control (i.e., “rest”) blocks. In the control task, the subjects were prompted to select the highest of four numbers presented instead of the cards. Each trial, consisting of one selection of four cards or numbers, lasted 4 s and each block consisted of 20 trials. Therefore, there was a total of 100 IGT trials in each scanning session. In the POST condition, the standard ABCD version of the task was substituted with a variant, known as EFGH, to minimize the learning effect (Bechara and Damasio, 2002; Bechara et al., 2002; Hernandez et al., 2006) and thus to reduce possible variation in brain activity between the PRE and POST conditions. The two versions differ with respect to the timing of losses and gains, so that while in the ABCD version gains are immediate and the losses are delayed, this situation is reversed for the EFGH variant. Subjects were told, before starting the EFGH task, that the strategy may differ to the previously completed task.

### Behavior

Subjects completed a battery of questionnaires both before and following stimulation with either the H-Coil, the Figure-8 coil, or the sham coil (*n* = 9, 8, and 12, respectively). The questionnaires were identical to those used in the feasibility study (see Extended Data Fig. 6-1 for a description). They included the mVAS questionnaire, which assesses motivation to undertake various actions (Stubbs et al., 2000; Gorwood et al., 2015); the AGQ, which evaluates two goal orientations toward a specific reward (Elliot and Sheldon, 1997); and the PANAS, which assesses emotional state (Watson et al., 1988). Subjects were asked to answer the items in the questionnaires according to how they felt at that moment to prevent, to as great an extent as possible, any effects of expectation or learning in the POST condition. In addition, all questionnaires were administered in writing and subjects were told that the data would be anonymized, to reduce social bias. The questionnaires were always administered to the subjects in the same order (mVAS→AGQ→PANAS).

10.1523/ENEURO.0287-22.2023.f6-1 Extended Data Figure 6-1 Description of behavioral questionnaires Download Figure 6-1, DOCX file.

### Statistics and data analysis

#### Neuroimaging

Image preprocessing and analysis were performed with the SPM8 software package (FIL), and the FSL package (fMRIB) was used for ROI drawing and data extraction.

The imaging data from each subject were first analyzed to identify areas of decreased and increased activation in the task blocks relative to the control (“rest”) blocks. The modeled fMRI signal was convolved with the hemodynamic response function, and low-frequency noise was removed with a high-pass filter (cutoff = 165 s). Group activation during the PRE IGT session was thresholded at *p* = 0.05 with voxel-level correction for familywise error rate (FWER). In the next stage, ROIs were drawn in the preselected areas described above and applied to the SPM contrast images containing the contrast of the parameter estimates at each voxel (contrast: task > rest) that had been obtained at the first-level in the PRE and POST images. The subsequent mixed model factorial ANOVA analyses investigated (1) the target ROI with a model comprising the factors TIME (within-subjects, two levels: PRE, POST) and COIL (between-subjects, three levels: H-Coil, Figure-8 coil, and sham), or (2) the reward-related ROIs with a model comprising the factors TIME (within-subjects, two levels: PRE, POST), COIL (between-subjects, three levels: H-Coil, Figure-8 coil, and sham) and ROI (within subjects, three levels: pgACC, right OFC, right insula). In order to establish the basis of the factorial ANOVA analysis, differences in the baseline (PRE) data between the coil groups were analyzed with one-way ANOVA tests and the normality assumption was assessed (Kolmogorov–Smirnov test on the residuals).

#### Behavior

Analysis was conducted using the SPSS statistical package (SPSS v20, IBM). In order to establish the basis of the factorial ANOVA analysis, the data were evaluated for normality and homogeneity of variances. Group differences in the baseline (PRE) data were analyzed with *t* tests. Unless stated otherwise, all effects are reported as significant at *p* ≤ 0.05. The effect of stimulation field distribution on the behavioral scores was analyzed with a two-way mixed 2 × 3 ANOVA design, with the factors TIME (PRE, POST) and COIL (H-Coil, Figure-8 coil, sham coil).

Decomposition of a significant interactions in factorial ANOVA analyses allows the origin of the finding to be explored in the framework of follow-up analyses. The approach of “simple effects” following significant findings and main effects following nonsignificant findings was employed (see Howell and Lacroix, 2012; their Fig. 1). Simple effects are commonly encountered in the form of simple main effects that are used to decompose a significant two-way interaction in which the effect of one independent variable is examined at individual levels of the other independent variable (Field, 2009). The equivalent approach following a significant three-way interaction is known as simple interaction effects (Howell and Lacroix, 2012). Effect sizes were calculated as partial η^2^ (η^2^_p_) for the main factorial analyses and as correlation coefficients for the single main effects analyses.

## Results

### Establishment of TMS protocol

High frequency stimulation of the right hemisphere was selected in the feasibility stage of the study since it was found to induce a greater degree of modulation of reward-motivated performance, corresponding to a general decrease in the behavioral scores (see Extended Data Figs. 6-2, 6-3). All subjects tolerated the rTMS well, and no serious adverse events were observed or reported. In addition, there were no significant differences between H-Coil and Figure-8 coil groups in self-reported ratings of comfort during stimulation (*t*_(15)_ = −0.58, *p* = 0.6^a1^). All statistics are summarized in Table 1.

**Table 1:** Statistical table

Line	Analysis (variables)	Type of test	Statistic	*p*-value and confidence
	Stimulation			
a1	Subject comfort (H-Coil vs Figure-8)	Unpaired *t* test	*t* = −0.58; DoF = 26	*p* = 0.56; CI = (−1.61,0.89)
	fMRI (BOLD signal)			
a2	fMRI (time, coil) for target ROI	Two-way ANOVA Main effect TIME Main effect COIL	*F* = 0.121; DoF = (2,17) *F* = 0.04; DoF = (1,17) *F* = 0.90; DoF = (2,17)	*p* = 0.9 *p* = 0.8 *p* = 0.4
a3	fMRI (time, coil, ROI)	Three-way ANOVA	*F* = 2.66; DoF = (4,34)	*p* = 0.49; η_p_^2^ = 0.25
a4	fMRI (time, coil)	Two-way ANOVA	*F* = 3.59; DoF = (2,17)	*p* = 0.50; η_p_^2^ = 0.30
a5	*Post hoc* on *a4* (OFC) H-Coil vs Figure-8 H-Coil vs sham	Simple main effect Simple main effect	Mean diff = 0.28 Mean diff = 0.31	*p* = 0.048^†^; CI = (0.00,0.55) *p* = 0.023^†^; CI = (0.05,0.57)
a6	*Post hoc* on *a4* (pgACC) H-Coil vs Figure-8 H-Coil vs sham	Simple main effect Simple main effect	Mean diff = 0.13 Mean diff = 0.13	*p* = 0.045^†^; CI = (0.00,0.26) *p* = 0.035^†^; CI = (0.01,0.26)
a7	*Post hoc* on *a4* (insula) H-Coil vs Figure-8 H-Coil vs sham	Simple main effect Simple main effect	Mean diff = 0.11 Mean diff = 0.11	*p* = 0.13^†^; CI = (−0.06,0.42) *p* = 0.26^†^; CI = (−0.10,0.36)
	fMRI (task performance)			
a8	IGT behavior (coil, time)	Two-way ANOVA	*F* = 0.86; DoF = (2,17)	*p* = 0.44
	Behavior (coil)			
a9	mVAS (time, coil)	Two-way ANOVA	*F* = 5.69; DoF = (2,26)	*p* = 0.009; η_p_^2^ = 0.3
a10	*Post hoc* on *a9* (H-Coil)	Simple main effect	*F* = 12.69; DoF = (1,27)	*p* = 0.001; *r*_2_ = 0.4
a11	ACQ (time, coil)	Two-way ANOVA	*F* = 2.86; DoF = (2,26)	*p* = 0.076; η_p_^2^ = 0.18
a12	*Post hoc* on *a11*	Main effect TIME Main effect COIL	*F* = 8.12; DoF = (1,27) *F* = 0.11; DoF = (2,27)	*p* = 0.01 *p* = 0.9
a13	PANAS (time, coil)	Two-way ANOVA	*F* = 0.28; DoF = (2,26)	*p* = 0.76; η_p_^2^ = 0.02
	Extended material		
	fMRI per ROI (*post hoc*)		
a14	OFC (coil, time)	Two-way ANOVA	*F* = 3.52; DoF = (2,17)	*p* = 0.048; η_p_^2^ = 0.30
a15	pgACC (coil, time)	Two-way ANOVA	*F* = 3.68; DoF = (2,17)	*p* = 0.047; η_p_^2^ = 0.31
a16	Insular cortex (coil, time)	Two-way ANOVA	*F* = 0.18; DoF = (2,17)	*p* = 0.18; η_p_^2^ = 0.18
	Behavior (frequency, side)		
a17	VAS (side, frequency, time)	Three-way ANOVA	*F* = 5.81; DoF = (1,35)	*p* = 0.021; η_p_^2^ = 0.15
a18	VAS (frequency, time)	Two-way ANOVA	*F* = 0.50; DoF = (1,35)	*p* = 0.04; η_p_^2^ = 0.01
a19	VAS (side, time)	Two-way ANOVA	*F* = 0.51; DoF = (1,35)	*p* = 0.51; η_p_^2^ = 0.01
a20	*Post hoc* on *a19*, low frequency	Two-way simple-interaction	*F* = 1.48; DoF = (1,35)	*p* = 0.23; *r*^2^ = 0.04
a21	*Post hoc* on *a19*, high frequency	Two-way simple-interaction	*F* = 4.76; DoF = (1,35)	*p* = 0.01; *r*_2_ = 0.12
a22	*Post hoc* on *a19*, right LPFC	2nd order simple main effects	*F* = 22.2; DoF = (1,17)	*p* = 0.001; *r*_2_ = 0.56
a23	ACQ (side, frequency, time)	Three-way ANOVA	*F* = 0.12; DoF = (1,35)	*p* = 0.73; η_p_^2^ = 0.10
a24	ACQ (frequency, time)	Two-way ANOVA	*F* = 4.19; DoF = (1,35)	*p* = 0.048; η_p_^2^ = 0.11
a25	ACQ (side, time)	Two-way ANOVA	*F* = 1.27; DoF = (1,35)	*p* = 0.27; η_p_^2^ = 0.04
a26	*Post hoc* on *a22*, high frequency	Simple main effect	*F* = 12.2; DoF = (1,17)	*p* = 0.001; *r*^2^ = 0.64
a27	PANAS (side, frequency, time)	Three-way ANOVA	*F* = 0.02; DoF = (1,35)	*p* = 0.90; η_p_^2^ = 0.00
a28	PANAS (frequency, time)	Two-way ANOVA	*F* = 1.90; DoF = (1,35)	*p* = 0.18; η_p_^2^ = 0.05
a29	PANAS (side, time)	Two-way ANOVA	*F* = 0.35; DoF = (1,35)	*p* = 0.56; η_p_^2^ = 0.00

All data were checked for normal distribution (see Materials and Methods, Statistical data and analysis).

CI: confidence interval.

DoF: degrees of freedom.

IGT: Iowa Game Task.

mVAS, ACQ, PANAS: behavioral questionnaires.

OFC, pgACC, insular: regions of interest.

† uncorrected *p*-value (multiply *p*-value by three for Bonferroni correction).

10.1523/ENEURO.0287-22.2023.f6-2 Extended Data Figure 6-2 Graphical results of the preliminary feasibility study, corresponding to the statistical findings in Extended Data Figure 6-3. Behavioral changes in motivational scores induced by the different combinations of stimulation side and frequency. The boxplot summarizes the changes in behavioral scores defined as PRE-POST of the mVAS score (***A***) or AGQ (***B***). A higher value thus represents a greater decrease in motivation following the stimulation. In each box, the horizontal band indicates the group median, the dot indicates the group mean, and the whiskers define the extent of 1.5 times the interquartile range. *p < 0.05. Download Figure 6-2, TIF file.

**Figure 3.**
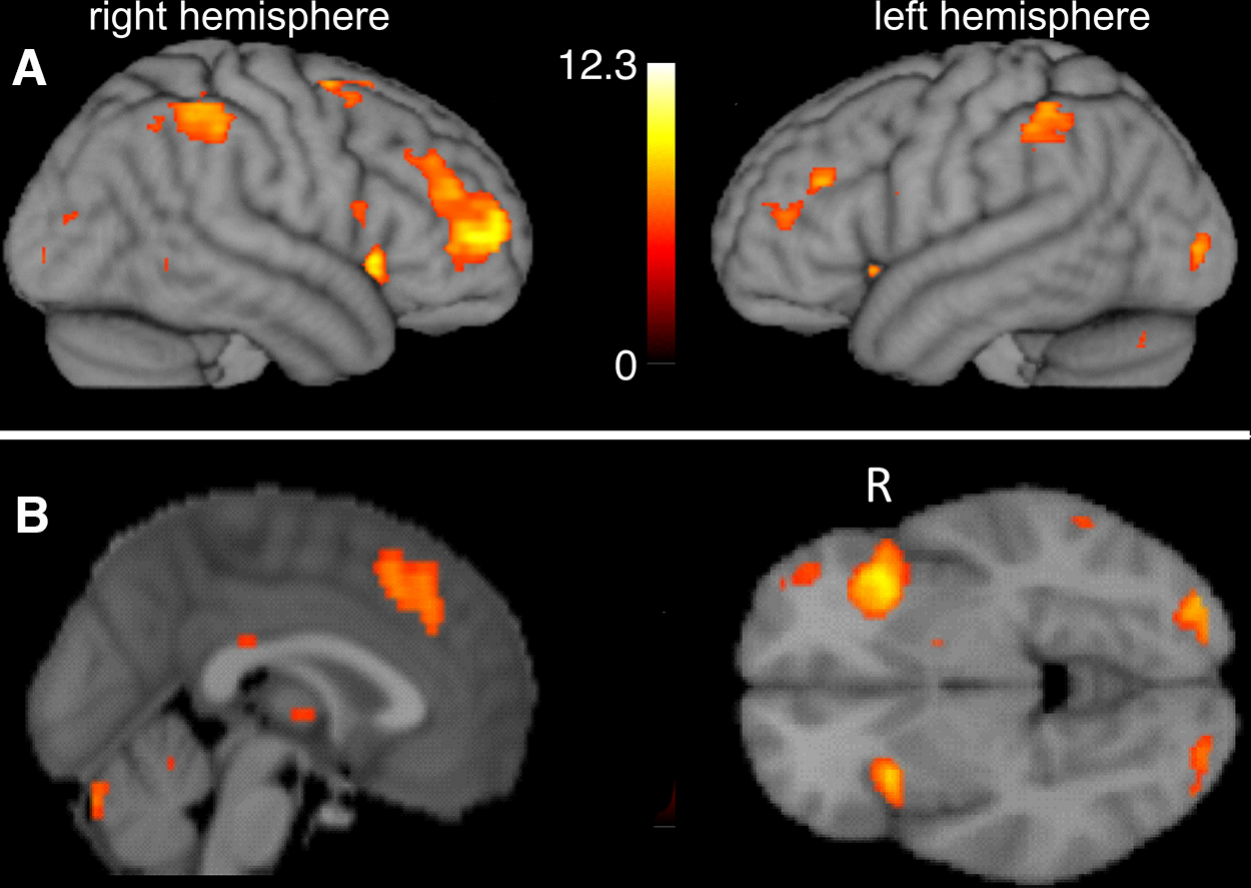
Group activation maps obtained while performing the IGT in the PRE (i.e., prestimulation) condition. Statistical maps (task > rest) are overlaid on a rendered cortical surface (***A***) or orthogonal slices (***B***) for a threshold of *p* = 0.05 with FWER correction. (L/R indicates left/right hemisphere in ***A*** and left/right sides of the image in ***B***, respectively.)

10.1523/ENEURO.0287-22.2023.f6-3 Extended Data Figure 6-3 Results of the preliminary feasibility study that was carried out in order to select the stimulation laterality and frequency that most robustly affect short-term motivational behavior. Thirty-nine subjects were randomly assigned to four groups in a two-way factorial design [1- or 10-Hz stimulation frequency, and right or left LPFC stimulation; subject numbers (*n*) = 11, 9, 9, 10 for the 1 Hz left, 10 Hz left, 1 Hz right, and 10 Hz right groups, respectively]. All groups were stimulated using the H-Coil, and the optimal frequency and site stimulation parameters were evaluated based on the results of questionnaires filled out both PRE and POST stimulation. TMS sessions lasted approximately 15 min and consisted of either high-frequency rTMS (10 Hz, 2-s trains, 20-s intertrain interval) or low-frequency rTMS (1 Hz, continuous train). In each session, 900 pulses were administered at an intensity of 120% of RMT, determined as the minimum stimulation output that induced 50% chance of visual thumb abduction (Pridmore et al., 1998). As in the main study, the target area was defined as the region 6 cm anterior to the primary motor hand area (M1) of the stimulated hemisphere. The effect of stimulation frequency and stimulation side on the behavioral scores was analyzed with a three-way mixed design, a 2 × 2 × 2 ANOVA with the factors TIME (PRE and POST), FREQUENCY (1 and 10 Hz), and SIDE (left and right DLPFC). The results are shown graphically in Extended Data Figure 6-1. The table reveals that analysis of the mVAS scores revealed a significant TIME × FREQUENCY × SIDE interaction. A two-way, simple-interaction follow-up analysis showed that the factor that drove this interaction was the 10-Hz stimulation (*F*_(1,35)_ = 1.484, *p* = 0.231, *r*^2^ = 0.04 ^a19^ and *F*_(1,35)_ = 4.764, *p* < 0.05, *r*^2^ = 0.12^a20^ for the simple TIME × SIDE interaction at the 1- and 10-Hz frequencies, respectively). Further decomposition of the simple interaction result revealed that it was driven by stimulation of the right LPFC, where a significant reduction was observed in the motivation of the subjects as assessed by the mVAS score (*F*_(1,17)_ = 22.2, *p* < 0.05, *r*^2^ = 0.56^a21^ for the second order simple main effects analysis). Decomposition of the three-way interaction starting with the alternative pathway of the simple TIME × FREQUENCY interaction, led to the same conclusions. Analysis of the AGQ score indicated a similar pattern of stimulation-induced changes in motivation but the three-way interaction was not significant. The next stage of follow-up analysis that examines the different “classical” two-way interactions (Howell and Lacroix, 2012), revealed a significant two-way TIME × FREQUENCY interaction (*p* < 0.05, η^2^_p_ = 0.11^a23^). Similar to the VAS analysis, decomposition of this result with a simple main effect revealed that the stimulation-induced changes in the AGQ score were significant only at 10 Hz (*F*_(1,17)_ = 12.2, *p* < 0.05, *r*^2^ = 0.64^a28^). Analysis of the PANAS^1^ scores revealed no significant interactions. Note that for brevity, only the positive-PANAS score are shown; the negative-PANAS scores were very similar in behavior. Measurements in the table are shown with SDs in brackets. FREQUENCY is abbreviated to FREQ in row headings. * indicates a significant effect; n.s. indicates a nonsignificant result. Download Figure 6-3, DOCX file.

Average stimulus output intensities at threshold were 62 ± 10% and 68 ± 8% for the Figure-8 coil and H-Coil, respectively. Differences in the stimulation intensity at threshold are expected because of the different coil designs. However, it is important to note that by calibrating the rTMS stimulus intensity in the standard manner according to the subject’s threshold, the study thereby is evaluating the effect of the different field distributions of the coils (Fig. 2) after fixing the strength of the electric field at the level of the hand motor cortex.

### Neuroimaging

Subjects were stimulated with either the H-Coil, Figure-8 coil, or sham coil. The data of one subject from the Figure-8 coil group were excluded because of head motion exceeding 2 mm that was observed during preprocessing. No differences were observed between the groups with regards to IGT performances, at both the PRE and POST conditions.

The group activation map obtained during the PRE task from all the subjects (i.e., *n* = 20) is shown overlaid on the cortical surface and orthogonal slices (Fig. 3*A*,*B*). Areas commonly included in the task-positive network such as the DLPFC and insula are observed.

**Figure 4.**
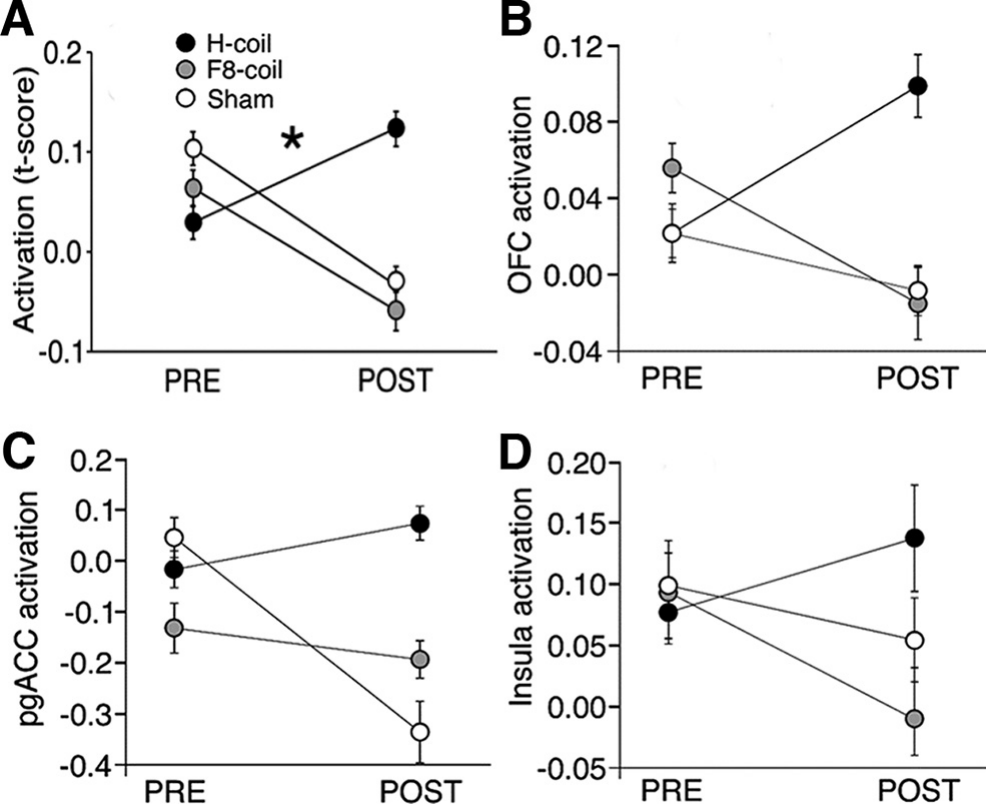
***A***, Effect of stimulation group on functional activation from the significant interaction (TIME × COIL) from the complete factorial model (TIME × COIL × ROI). Activation, shown on the *y*-axis, is represented by the SPM contrast image for task > rest. The plots thereby indicate the stimulation-induced activation for the three stimulation groups over the two time points (PRE and POST) and the corresponding slopes characterize the sign of this activation-induced change. The ROIs were in areas of the putative reward network, in the right OFC, right insula, and pgACC. Extended Data Figure 4-1 in the extended material shows the corresponding plot for changes in the behavioral performance metric measured during the IGT task. ***B–D***, Exploratory analysis of TIME × COIL interaction in each of the individual ROIs, the right orbitofrontal cortex (***B***), the pregenual ACC (***C***), and the right insular cortex (***D***). The same pattern of opposing slopes shown in ***A*** is consistently observed in these individual ROIs that represented a nonsignificant trend following correction for multiple comparisons (right OFC ROI: *F*_(2,17)_ = 3.52, *p*_uncorrected_ = 0.048, *p*_corrected_ = 0.14, η^2^_p_ = 0.30^a13^; pgACC ROI: *F*_(2,17)_ = 3.68, *p*_uncorrected_ = 0.047, *p*_corrected_ = 0.14, η^2^_p_ = 0.31^a14^; right insular cortex: *F*_(2,17)_ = 1.9, *p*_uncorrected_ = 0.18, *p*_corrected_ = 0.54, η^2^_p_ = 0.18^a15^). Errors bars are ±SEM, * indicates a significant interaction (*p* < 0.05).

In the mixed model ANOVA, no statistical differences were observed in the baseline (i.e., PRE) data across the coil groups and normality of residuals was confirmed. In the analysis of the target ROI, no significant terms were found either in the interaction or main effects (TIME × COIL: *F*_(2,17)_ = 0.121, *p* = 0.9; TIME: *F*_(1,17)_ = 0.04, *p* = 0.8; COIL: *F*_(2,17)_ = 0.90, *p* = 0.4^a2^) indicating that the task did not differentially activate this region between the stimulation states. In the analysis of the task-related ROIs, the three-way interaction (TIME × COIL × ROI) was found to be significant (*F*_(4,34)_ = 2.66, *p* = 0.049, partial η^2^ = 0.25^a3^) indicating two-way interactions that vary across levels of the third variable. The TIME × COIL term was also significant (*F*_(2,17)_ = 3.59, *p* = 0.05, partial η^2^ = 0.30^a4^) which is suggestive of a consistent influence of the coil on the stimulation-induced activation regardless of the particular ROI. The plot of TIME × COIL interaction was visually characterized by opposing sign of the “slope” (i.e., POST-PRE activation-induced change) for the H-Coil stimulation group compared with the two other stimulation groups (Fig. 4*A*). Follow-up analysis of the TIME × COIL interaction plots at each level of ROI consistently indicated this same visual pattern of opposing slopes (see Fig. 4*B–D*). Evaluation of these simple main effects of the TIME × COIL term confirmed that in two of the three ROIs, the poststimulation activation for the H-Coil displayed a trend of difference relative the Figure-8 and sham coils (for example, in the right orbitofrontal cortex ROI, H-Coil vs Figure-8 mean difference = 0.23 ± 0.13. *p*_uncorrected_ = 0.045; H-Coil vs sham mean difference = 0.31 ± 0.13. *p*_uncorrected_ = 0.05^a5,a6,a7^).

10.1523/ENEURO.0287-22.2023.f4-1 Extended Data Figure 4-1 The behavioral performance metric measured during the IGT task performed in the MRI scanner. The metric is defined by calculating a net score in each block, defined as the difference between the number of selections from the advantageous (ABCD) and disadvantageous (EFGH) decks. The metric did not differ between coil interventions, and the interaction was not significant (two-way ANOVA, *F*_(2,17)_ = 0.864, *p* = 0.44^a6^). However, it is interesting to note that the pattern of interstimulation slopes for this metric was similar to that observed for the neuroimaging ROIs (see Fig. 4). Download Figure 4-1, TIF file.

The behavioral performance metric measured during the IGT task did not significantly differ between stimulation groups (*F*_(2,17)_ = 0.864, *p* = 0.44^a7^; see Extended Data Fig. 4-1).

**Figure 5.**
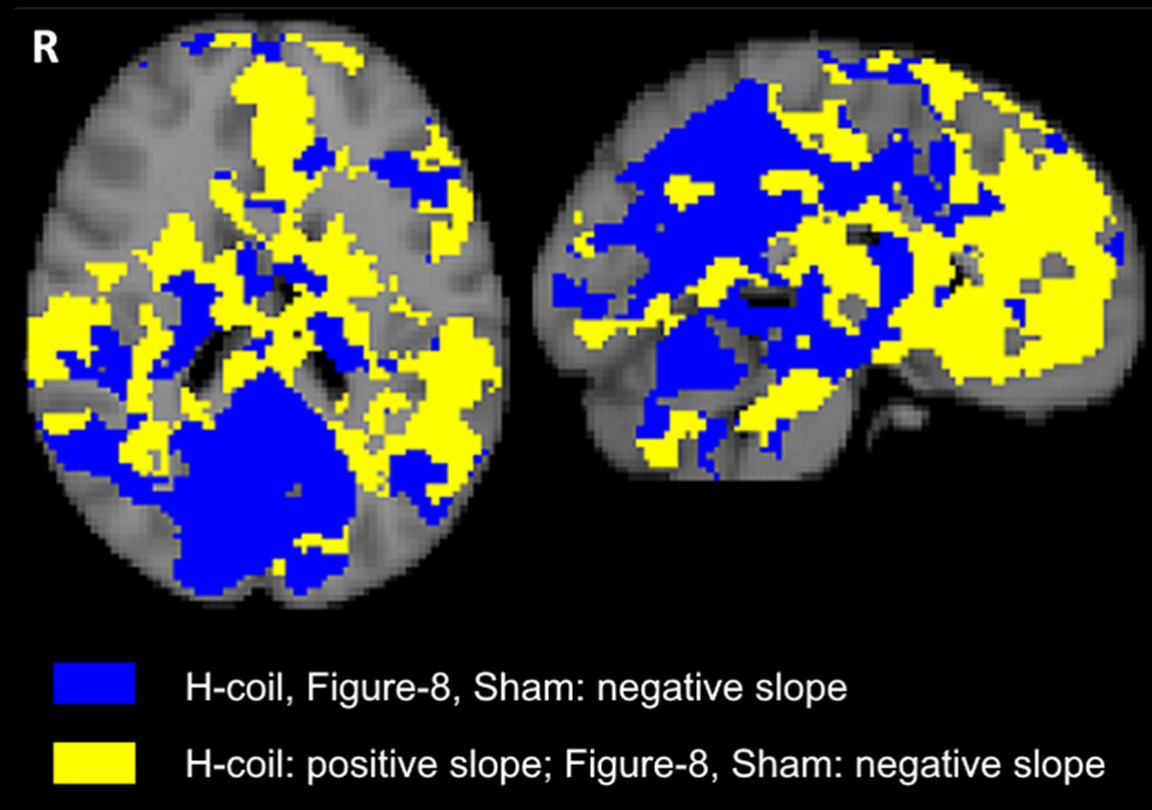
Explorative analysis showing intercoil pattern of slopes, defined as the change in the activation contrast of task > rest between the POST and PRE scans, i.e., as POST-PRE. Two representative orthogonal slices are shown with Talairach slice positions: axial (left): z = 20 cm; sagittal (right): x = −4 cm. Yellow voxels represent the stereotypical pattern of activation seen across the three selected ROIs in the reward system, i.e., with a positive slope in the H-Coil stimulation group and a negative slope in the Figure-8 coil and Sham stimulation groups. Blue voxels represent the nondifferential pattern of activation (namely, negative slopes for all stimulation groups).

To further explore the spread of this signal pattern, a voxel-wise explorative analysis was conducted across the brain, to reveal areas in which this phenomenon of opposing activation-induced slope was observed even in the absence of a significant statistical interaction. The analysis found the pattern to be repeated in widespread areas of the brain, with a clear division along the anterior-posterior axis of the brain (Fig. 5). That is, anterior areas were largely characterized by a positive slope of activation-induced change (i.e., POST>PRE) following H-Coil stimulation and negative slope following Figure-8 coil or Sham stimulation, while posterior areas were largely characterized by negative slopes (i.e., PRE>POST) across all stimulation conditions.

### Behavior

**Figure 6.**
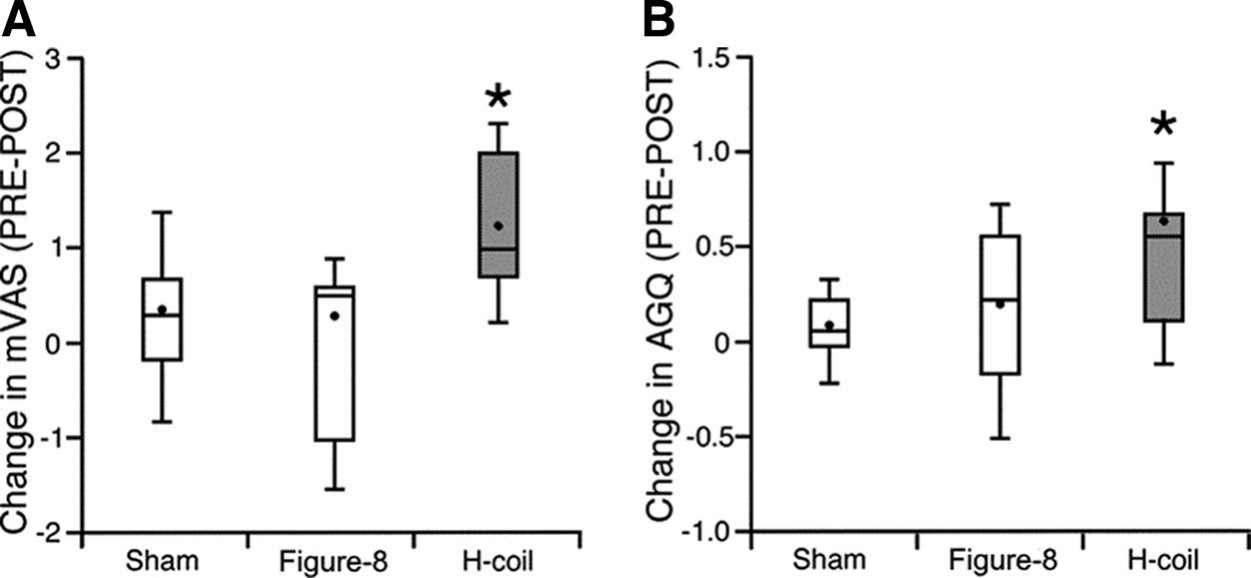
Behavioral changes in motivation induced by the various stimulation coils (defined as PRE-POST). In each box of the mVAS (***A***) and AGQ (***B***) scores as measured by the questionnaires, the horizontal band indicates the group median, the dot indicates the group mean, and the whiskers define the extent of 1.5 times the interquartile range. Extended Data Figure 6-1 in the Extended Data shows detailed explanations of the questionnaires. Extended Data Figures 6-2 and 6-3 show analyses of these measures obtained in the preliminary feasibility study. *p < 0.05 between PRE and POST H-Coil stimulation.

No statistical differences in the baseline (PRE) data between the coil groups were observed for any of the behavioral measures and normality of residuals was confirmed. Following high frequency stimulation of the right PFC, analysis of the mVAS scores revealed a significant TIME × COIL interaction (*F*_(2,26)_ = 5.69, *p* = 0.009, η^2^_p_ = 0.3 ^a8^; see Fig. 6*A*), and decomposition for simple main effects revealed significantly decreased score only following H-Coil stimulation (*F*_(1,27)_ = 12.69, *p* = 0.001, *r*^2^ = 0.4^a9^), but not following Figure-8 coil or Sham stimulation, thereby indicating that it was the behavior of the H-Coil that drove the interaction. Analysis of the AQG scores indicated a similar qualitative pattern. In this case however, the TIME × COIL interaction did not reach significance (*F*_(2,27)_ = 2.86, *p* = 0.076, η^2^_p_ = 0.18; Fig. 6*B*)^a10^. The main effect of TIME was significant (*F*_(1,27)_ = 8.12, *p* = 0.01), while the main effect of COIL was not significant (*F*_(2,27)_ = 0.11, *p* > 0.05)^a11^. Exploratory analysis of simple main effects again indicated significantly decreased score following H-Coil stimulation (*F*_(1,27)_ = 12.21, *p* = 0.002, *r*^2^ = 0.3^a11^), but not following Figure-8 coil or Sham stimulation. Analysis of the PANAS scores revealed no significant interactions (*F*_(2,26)_ = 0.28, *p* = 0.76, η^2^_p_ = 0.02^a12^) or simple main effects.

## Discussion

This is the first study that compares patterns of activation that are generated following stimulation with the Figure-8 coil and the H-Coil. It was found that right-sided stimulation at high-frequency led to reduced behavioral scores and a unique pattern of activation changes following H-Coil, but not Figure-8 coil or Sham stimulation. Taken together with the more focal spatial distribution of the electric field induced by the Figure-8 coil and the lack of coil-related differences in the target ROI, these results suggest that the origin of the observed effects is outside the area in the DLPFC conventionally targeted during TMS treatment, but inside the wider stimulation area of the H-Coil. Future experiments may identify an exact location in the wider LPFC, or even in more remote areas such as the medial PFC. Alternatively, it is possible that the effect is generated in deeper layers of the DLPFC that are beyond the reach of the Figure-8 coil.

While the study has demonstrated the ability of TMS to modulate motivational behavior, the reduction in these measures observed with stimulation in the chosen PFC target is evidence of the complex relationship of brain stimulation and cognitive function where “improvements” in function are often hard to achieve because of the complex brain networks involved (Sack and Linden, 2003).

The feasibility stage of the study selected high frequency stimulation of the right hemisphere as it was found to induce a greater degree of modulation of reward-motivated performance. Interestingly, this was found to be the optimal protocol for treatment of attention-deficit disorder (ADHD) using the same coil, presumably because of its ability to effectively reduce response inhibition and reward-based decision-making (Alyagon et al., 2020). Left-sided stimulation surprisingly did not induce a corresponding increase in performance which might reflect the difficulty in inducing such a change in healthy subjects. The decrease in motivation, as assessed by the mVAS and AGQ questionnaires, following right-sided stimulation appears to be in accordance with the theory of hemispheric specialization of the PFC (Davidson, 1992; Heller et al., 1995; Coan and Allen, 2004; Pizzagalli et al., 2005; Spielberg et al., 2012) and the rationale for DLPFC stimulation for the treatment of depression. That is, while high-frequency stimulation of the left DLPFC is expected to alleviate the anhedonic and amotivational symptoms of depression, stimulation of its contralateral homolog is expected to do the opposite. In our healthy volunteers, where the hedonic and motivational states are not compromised, a ceiling effect may have prevented additional improvement following left stimulation, but impaired motivation following right stimulation.

The effect of stimulation on reward-related behavior was significant only for the more widespread stimulation with the H-Coil. This may follow the simple consequence that coils with a broader field profile will be more likely to overlap with the location of the optimal target region; alternatively, stimulation of multiple, deeper or more downstream targets, may also play a role. For example, connectivity-based targeting (Fox et al., 2012) showed that the efficiency of rTMS treatment of depression is related to the strength of the connectivity between the stimulated region within the DLPFC and the subgenual ACC (sgACC), a deep brain structure which has an important role in the reward circuitry.

While the Figure-8 coil and Sham stimulation did not affect motivational behavior performance, the H-Coil lead to reduced score in the “pure” motivational questionnaire (mVAS), intermediate reduction of scores in the questionnaire that combine motivational and affective influences (AGQ) and did not affected scores in the “pure” affective questionnaire (PANAS). These results suggest that H-Coil stimulation influenced the cognitive, rather than the affective, aspects of goal-oriented behavior, a result that is line with former publications (Jenkins et al., 2002; Levkovitz et al., 2007). However, an alternative interpretation is that the effect of stimulation decayed faster than expected, and the degree of influence over the different questionnaires is because of the order of their administration (i.e., mVAS→AGQ→PANAS).

The neuroimaging data confirmed the influence of the spatial field distribution by showing that stimulation-induced change in fMRI activation across several regions in the reward network displayed a unique behavior in the group that received stimulation with the H-Coil. This corresponded to the observation of a characteristic pattern in the plots of PRE versus POST activation. On further examination, this pattern was predominantly in anterior areas of the brain, within areas commonly associated with both the Task Negative network (e.g., dACC and ventromedial PFC) and the Task Positive network (e.g., insula and ventrolateral PFC).

A recent study by Siddiqi et al. (2020), showing that focal TMS affects distinct aspects of MDD symptoms depending on coil location, suggests the utilization of coils with less focality to potentially provide symptom-wide benefits. Our study has shown that combinations of coils with different spatial characteristics can be used to aid our understanding of the anatomic origin of complex cognitive and behavioral processes.

We acknowledge several limitations to this study, principally the relatively small sample sizes. While the hypothesis-driven imaging analysis was restricted to ROIs in the reward system, a data-driven whole-brain voxel-wise analysis revealed a pattern of signal changes that was replicated across widespread areas of the brain. However, discussion related to the nonsignificant findings is speculative. Finally, it should be noted that neuro-navigation was not used in this study, which may have influenced the results of focal stimulation.

In conclusion, this study used distinct stimulation profiles of TMS coils to investigate the manipulation of reward-related behavior and neuronal function. This approach was shown to represent a promising tool to explore the regional specificity of behavior. The study thus supports the use of different TMS coil types and TMS targets to enhance our understanding of human behavior. These findings contribute to the growing knowledge of the neurobiology of cognition and may provide the basis of innovative protocols for the controllable modulation of reward-oriented behavior.

## Synthesis

Reviewing Editor: Anne Keitel, University of Dundee Decisions are customarily a result of the Reviewing Editor and the peer reviewers coming together and discussing their recommendations until a consensus is reached. When revisions are invited, a fact-based synthesis statement explaining their decision and outlining what is needed to prepare a revision will be listed below. The following reviewer(s) agreed to reveal their identity: Domenica Veniero.

As you know, it was difficult to find a sufficient number of reviewers, despite our best efforts and contacting dozens of individuals. To be able to come to a decision regarding your manuscript, we had an editorial consultation, in addition to the comments received by one reviewer. We agreed that the topic is very interesting from a methodological perspective and there seem to be no major flaws in the experimental procedures or statistical analyses.

Please find below the unabridged comments by reviewer #1, to allow you to address these in a point-by-point manner in a revised version of the manuscript.

Reviewer #1 - Advances the Field (Required)

The study described in the manuscript provides interesting information for the brain stimulation community. Specifically, it addressed an interesting question on whether decreasing the focality of the stimulation leads to an advantage compared to more classical protocols where focality is considered the main advantage of TMS.

Reviewer #1 - Statistics

Some sections were unclear. I think the authors should explain better why some tests were run. I don’t think it’s a major flaw of the paper.

Reviewer #1 - Extended Data

Extended data report complementary findings which are fundamental to understanding and evaluating the main manuscript.

Reviewer #1 - Comments to the Authors (Required)

In this paper, the authors evaluate the effect of high-frequency TMS delivered over the left prefrontal cortex. To this aim, they evaluate changes in brain activity as measured during an fMRI study and collect the score to several questionnaires that evaluate motivation and affect. The interesting manipulation that they add to the study is the electrical field distribution which they change by employing either a classical figure-of-8 coil or an H-coil. They found some changes post-stimulation in brain activity and in motivational scores.

I think the study is interesting, nonetheless, I have some questions about the theoretical framework and some statistical comparisons. Some sections of the paper were slightly unclear to me.

In the introduction, the authors seem to relate the aim of the study to previous results obtained in clinical populations and put forward the problem of focality when applying TMS to patients. They seem to conclude that the H-coil, which is known to stimulate wider and deeper areas of the brain, might overcome the issue of finding a specific target that would modulate only some of the symptoms. This is quite a big part of the introduction, but I am not sure how this relates to their results in particular to a decreased motivational score after the stimulation.

Related to this point, the conclusion of the introduction is that this study will provide findings that can shed light on regional specificity within the prefrontal cortex. However, as far as I can understand the aim is not functionally mapping different regions of the prefrontal cortex but rather testing the efficacy of the H-coil which is characterised by a reduced focality. This seems to be confirmed by the Siddiqi et al paper that is cited in support of the idea that a less focal coil should lead to a more effective protocol to stimulate the prefrontal cortex in patients.

I would suggest adding a brief explanation of the behavioural scores, for readers who are not familiar with the questionnaires used here, e.g., what is the meaning of a higher score on the mVAS?

I am not sure I understand the logic behind the statistics performed on the neuroimaging data. In the results, the authors report a significant Time x Coil x ROI interaction. This would allow them to perform follow-up ANOVAs Time x Coil for each ROI separately. However, they only perform follow-up analysis on Time x Coil. Why is the exploratory analysis needed?

On the other hand, for the AQG scores, they report no significant TIME×COIL interaction was found and yet they proceed with follow-up tests.

It would be important to report for each significant effect found for the H-coil, whether it is also different from the sham and f8 coil.

H-coil stimulation decreases motivational scores, how does this relate to patient studies/usefulness in patient populations?

Minor points:

Figure 4-2. The legend should be consistent with the others and report coil types rather than regions.

It is my understanding that all parameters for the stimulation in the main experiment relied exclusively on the questionnaire scores, not the IGT. Is this correct?

Is the section titled TMS protocol all about the feasibility study? This is unclear and I would suggest renaming the section if this is the case.

I could not find the results of the sensation questionnaire.
