# Supplementary material for: Behavioral and Functional Brain Activity Alterations Induced by TMS Coils with Different Spatial Distributions
Source: eNeuro. 2023 Apr 12;10(4):ENEURO.0287-22.2023. doi: 10.1523/ENEURO.0287-22.2023 (PMC10112547; doi:10.1523/ENEURO.0287-22.2023)
Supplement: Extended Data Figure 6-1 — Description of behavioral questionnaires Download Figure 6-1, DOCX file. [file enu-eN-NWR-0287-22-s05.docx]

Figure 6-1 Description of questionnaires and scales used in this study

| **Questionnaire** | **Description** |
| --- | --- |
| **mVAS** | The Motivational Visual Analog Scale (mVAS) questionnaire designed for this study to assess motivation was based on the principles of the visual analogue scale (hence, denoted mVAS). A similar approach to assess motivation was shown to be highly reliable in repeated measures designs (Stubbs et al., 2000). By drawing a pencil mark on 10 cm long analogue scales, subjects were asked to rate their motivation to undertake various actions: (1) listen to their favorite music, (2) solve a logical puzzle, (3) meet your favorite friend, and (4) climb 1000 stairs for 100 NIS (approximately $25). The average score across these questions was calculated to define the overall result. As a control measure, the subjects were asked to rate their general feeling and desire to participate in the experiment. |
| **ACQ** | The Achievement Goal Questionnaire (AGQ), designed to distinguish between different goal orientations towards a specific reward, was adapted from a previous study (Elliot and Sheldon, 1997) by replacing the focus of motivation from the desire to complete a long university course to the desire to succeed in a short comprehension test. The rationale for this choice was based on the notion that the effects of single stimulation session are transient, and that the task should correspond with that short timing. The subjects were told that, at the end of the experiment, they will need to read an article in a topic of their choice and that a series of questions will then be used to test their reading comprehension. They were then presented with the questions taken from the original AGQ reference (Elliot and Sheldon, 1997) to assess their motivation to succeed in the comprehension test. The results from the different AGQ categories were pooled together to provide an overall score. |
| **PANAS** | In the Positive and Negative Affect Schedule (PANAS) questionnaire that assesses emotional state, the subjects were asked to rate how a list of feelings describes their inner mental state (Watson et al., 1988). For each feeling, the subjects were requested to rate between 1-5 how the feeling characterizes their emotions at the present moment. Both positive and negative mood scales were evaluated (positive and negative PANAS, respectively). |
