# Supplementary material for: Behavioral and Functional Brain Activity Alterations Induced by TMS Coils with Different Spatial Distributions
Source: eNeuro. 2023 Apr 12;10(4):ENEURO.0287-22.2023. doi: 10.1523/ENEURO.0287-22.2023 (PMC10112547; doi:10.1523/ENEURO.0287-22.2023)
Supplement: Extended Data Figure 6-3 — Results of the preliminary feasibility study that was carried out in order to select the stimulation laterality and frequency that most robustly affect short-term motivational behavior. Thirty-nine subjects were randomly assigned to four groups in a two-way factorial design [1- or 10-Hz stimulation frequency, and right or left LPFC stimulation; subject numbers (n) = 11, 9, 9, 10 for the 1 Hz left, 10 Hz left, 1 Hz right, and 10 Hz right groups, respectively]. All groups were stimulated using the H-Coil, and the optimal frequency and site stimulation parameters were evaluated based on the results of questionnaires filled out both PRE and POST stimulation. TMS sessions lasted approximately 15 min and consisted of either high-frequency rTMS (10 Hz, 2-s trains, 20-s intertrain interval) or low-frequency rTMS (1 Hz, continuous train). In each session, 900 pulses were administered at an intensity of 120% of RMT, determined as the minimum stimulation output that induced 50% chance of visual thumb abduction (Pridmore et al., 1998). As in the main study, the target area was defined as the region 6 cm anterior to the primary motor hand area (M1) of the stimulated hemisphere. The effect of stimulation frequency and stimulation side on the behavioral scores was analyzed with a three-way mixed design, a 2 × 2 × 2 ANOVA with the factors TIME (PRE and POST), FREQUENCY (1 and 10 Hz), and SIDE (left and right DLPFC). The results are shown graphically in Extended Data Figure 6-1. The table reveals that analysis of the mVAS scores revealed a significant TIME × FREQUENCY × SIDE interaction. A two-way, simple-interaction follow-up analysis showed that the factor that drove this interaction was the 10-Hz stimulation (F(1,35) = 1.484, p = 0.231, r2 = 0.04 a19 and F(1,35) = 4.764, p < 0.05, r2 = 0.12a20 for the simple TIME × SIDE interaction at the 1- and 10-Hz frequencies, respectively). Further decomposition of the simple interaction result revealed that it was driven by stimulation of t [file enu-eN-NWR-0287-22-s04.docx]

Figure 6-3. Summary and statistical analyses of the behavioral measures in the preliminary feasibility study evaluated PRE and POST stimulation.

|  | | **PRE, mean (SD)** | **POST, mean (SD)** |
| --- | --- | --- | --- |
| **VAS** | 1Hz, Left | 5.83 (1.84) | 5.15 (1.77) |
|  | 10Hz, Left | 5.55 (1.17) | 5.36 (1.34) |
|  | 1Hz, Right | 5.35 (1.20) | 5.16 (1.44) |
|  | 10Hz, Right | 5.82 (1.30) | 4.71 (1.41) |
|  | TIME×FREQ×SIDE | F(1,35)=5.818, p=0.021*, η^2^_p_=0.14 ^a16^ | |
|  | TIME×FREQ | F(1,35)=0.502, p=0.043, η^2^_p_=0.01 ^a17^ | |
|  | TIME×SIDE | F(1,35)=0.506, p=0.506, η^2^_p_=0.01 ^a18^ | |
| **AGQ** | 1Hz, Left | 3.43 (0.97) | 3.43 (1.19) |
|  | 10Hz, Left | 3.97 (1.05) | 3.60 (1.17) |
|  | 1Hz, Right | 4.07 (0.88) | 3.90 (1.24) |
|  | 10Hz, Right | 4.24 (0.71) | 3.57 (1.02) |
|  | TIME×FREQ×SIDE | F(1,35)=0.122, p=0.729, η^2^_p_=0.00 ^a22^ | |
|  | TIME×FREQ | F(1,35)=4.187, p=0.048*, η^2^_p_=0.11 ^a23^ | |
|  | TIME×SIDE | F(1,35)=1.273, p-0.267, η^2^_p_=0.04 ^a24^ | |
| **PANAS (positive)^1^** | 1Hz, Left | 3.01 (0.42) | 2.83 (0.76) |
|  | 10Hz, Left | 3.17 (0.39) | 2.79 (0.52) |
|  | 1Hz, Right | 3.04 (0.63) | 2.78 (0.58) |
|  | 10Hz, Right | 3.31 (0.38) | 2.82 (0.38) |
|  | TIME×FREQ×SIDE | F(1,35)=0.015, p=0.903, η^2^_p_=0.00 ^a25^ | |
|  | TIME×FREQ | F(1,35)=1.895, p=0.177, η^2^_p_=0.05 ^a26^ | |
|  | TIME×SIDE | F(1,35)=0.354, p=0.556, η^2^_p_=0.00 ^a27^ | |
