# Supplementary figures and images for: Behavioral and Functional Brain Activity Alterations Induced by TMS Coils with Different Spatial Distributions
Source: eNeuro. 2023 Apr 12;10(4):ENEURO.0287-22.2023. doi: 10.1523/ENEURO.0287-22.2023 (PMC10112547; doi:10.1523/ENEURO.0287-22.2023)

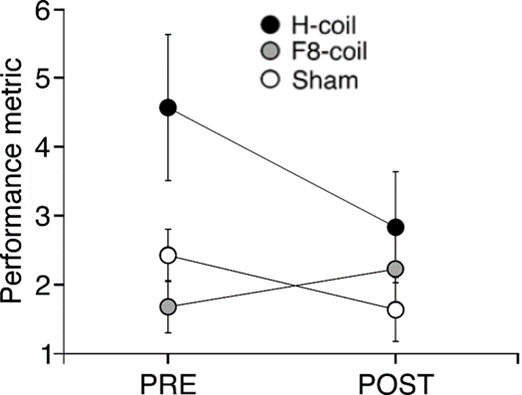

Supplement: Extended Data Figure 4-1 — The behavioral performance metric measured during the IGT task performed in the MRI scanner. The metric is defined by calculating a net score in each block, defined as the difference between the number of selections from the advantageous (ABCD) and disadvantageous (EFGH) decks. The metric did not differ between coil interventions, and the interaction was not significant (two-way ANOVA, F(2,17) = 0.864, p = 0.44a6). However, it is interesting to note that the pattern of interstimulation slopes for this metric was similar to that observed for the neuroimaging ROIs (see Fig. 4). Download Figure 4-1, TIF file. [file enu-eN-NWR-0287-22-s02.tif]
